# Aniplant or plantimal? Superorganisms cross borders

**DOI:** 10.1007/s00709-021-01726-x

**Published:** 2021-12-07

**Authors:** Peter Nick

**Affiliations:** Botanical Institute, Karlsruhe Institute of Technology, Karlsruhe, Germany

What is an organism? We tend to see organisms as bodies, composed of cells coming from the same origin and therefore sharing the same genome. It is getting progressively clear that this conception is an inappropriate reduction of a far more complex reality. While we know through the work of Mereshkowski (1905) and Margulis (1970) that already the building blocks of these bodies, cells, are functional, rather than genetic units, we are still far from appreciating that also organisms are not genetic, but functional units that often house life forms of completely different provenance. Thus, organisms are defined by the interactions between their components, at least to the same extent as they are shaped by their genes. Two contributions to the current issue highlight fascinating aspects of these interactions and allow watching new “superorganisms” in *statu nascendi*.

The work by Bragança et al. (2021) deals with insect galls, amazing structures induced by plant-parasitic insects by hijacking signalling of the host to produce an intricately structured new structure, which has the purpose to provide plant metabolites to the developing larvae. This must not only include signals that suppress plant immunity, but also signals that reorchestrate developmental processes of the host plant in a manner that a novel organ develops that is not foreseen in the genetic programme of the host. Using a system involving Cecidomyiidae as parasite and the Leguminose host *Inga ingoides*, they show that three types of galls exist that differ not only with respect to their cytological details, but also with respect to their metabolic profiles. Two of those, the ambrosia globoid type and the fusiform galls, involve death of host cells, while the third, termed lenticular, preserves viability of host cells. A closer look reveals that the cell death of the ambrosia globoid type differs from that in the fusiform galls and is actually linked with the presence of hyphae with peculiar cellular features such as a high density of multivesicular bodies. Thus, this type of galls is even reaching beyond an interaction of two organisms. Here, three organisms coming from different realms of life interact in a specific way and bring about a novel structure, a fine example that evolution can act across different streams of vertical gene flow.

In the work by Kodama and Sumita (2021) , the roles are inversed. Here, it is a plant that invades an animal host, for the sake of both sides. Green microalgae from the genus *Chlorella* can be ingested by the ciliate *Paramecium bursaria* and can escape digestive vacuoles and persist in the cytoplasm, enclosed in a specific perialgal vacuole. The benefit for the host is evident —in times of starvation, it can rely on a carbon source that is fed by light. The endophyte benefits from being carried around by a mobile carrier that ensures a sufficient supply of sunlight. This symbiosis is not obligatory since both partners can persist also in the non-associated state. In fact, it is possible to free the host from its plant inhabitants and to have it recolonised afterwards. Thus, this case is interesting because it allows observing early stages in the establishment of a superorganism. In their previous work (Kodama and Fujishima 2005), the authors had shown that the first step of uptake was not of a friendly type. The *Chlorella* cells are swallowed as any other prey. For instance, acidosomes and lysosomes with digestive enzymes fuse to produce a digestive vacuole. The difference occurs only in the second step, when the algal cell resists digestion and induces a bud of the digestive vacuole that is split off and differentiates into a perialgal vacuole that is able to ward off further lysosomes. The central point is how the endosymbiont is differentiated from a prey. The authors addressed this by feeding fluorescent microbeads mixed with real-world algal cells. They found that these microspheres were efficiently filtered out and did not end up in the perialgal vacuole. However, the rate, by which the endosymbionts were transferred into this symbiotic organelle, was significantly reduced in the presence of the microsphere. This means that the sorting is an active process of the host that is limited by a host-factor that is recruited either for digestion or for symbiosis. Thus, although the two organisms belong to different kingdoms of life and still enjoy full autonomy, the host has already evolved mechanisms to welcome the endosymbiont, and it invests parts of its digestive powers for the new task to integrate a cell originating from a different kingdom of life.

In both cases, the insect-induced galls and the ciliate *P. bursaria*, the host needs to invest something. The gall-forming plant concentrates on assimilating for the sake of its animal guest. The re-colonised ciliate offers parts of its digestive powers for the sake of generating perialgal vacuoles. As different as the “aniplant” and the “plantimal” may seem, they both sacrifice something for the sake of the ensuing co-habitation. However, at first sight, the two cases appear different because in the case of the galls the benefit seems to lie entirely on the side of the colonising animal, while in the case of the green ciliate both partners profit from the interaction. However, this judgement might be biased, since recent findings demonstrate that galled plants are more resilient to herbivory (Kurzfeld-Zexer and Inbar 2021). Thus, in both cases, sacrifice represents the first step towards integration.
